# Pharmaceutical Association

**Published:** 1878-10-20

**Authors:** 


### PHARMACEUTICAL ASSOCIATION.

The meeting of American Pharmaceutical As- hus been postponed to the 26th of November. It will convene in Atlanta Georgia.

A very full attendance is anticipated. Ample arrangements, we learn, will be made for the reception of the members.

